# RskA Is a Dual Function Activator-Inhibitor That Controls SigK Activity Across Distinct Bacterial Genera

**DOI:** 10.3389/fmicb.2020.558166

**Published:** 2020-09-09

**Authors:** Frédéric J. Veyrier, Cecilia Nieves, Louise H. Lefrancois, Hana Trigui, Antony T. Vincent, Marcel A. Behr

**Affiliations:** ^1^Bacterial Symbionts Evolution, Centre Armand-Frappier Santé Biotechnologie, Institut National de la Recherche Scientifique, Université du Québec, Laval, QC, Canada; ^2^McGill International TB Centre, Montreal, QC, Canada; ^3^Department of Medicine, McGill University, Montreal, QC, Canada

**Keywords:** *M. tuberculosis*, *M. bovis*, *M. orygis*, *Yersinia*, Sigma factor K, anti-Sigma factor, RskA

## Abstract

It has been previously shown that RskA, the anti-Sigma factor K of *Mycobacterium tuberculosis*, inhibits SigK and that mutations in RskA promote high expression of the SigK regulon. The latter observation led us to hypothesize that RskA mutations lead to loss of the anti-Sigma factor function. In this report, we used natural and artificial mutations in RskA to determine the basis of the SigK-RskA partnership. Consistent with predictions, the N-terminal cytoplasmic portion of RskA was sufficient on its own to inhibit SigK. Unexpectedly, RskA also served as an activator of SigK. This activation depended on the same N-terminal region and was enhanced by the membrane-extracellular portion of RskA. Based on this, we engineered similar truncations in a Gram-negative bacterium, namely *Yersinia enterocolitica*. Again, we observed that, with specific alterations of RskA, we were able to enhance SigK activity. Together these results support an alternative mechanism of anti-Sigma factor function, that we could term modulator (activator-inhibitor) in both *Actinobacteria* and Gram-negative bacteria, suggesting that Sigma factor activation by anti-Sigma factors could be under-recognized.

## Introduction

The *Mycobacterium tuberculosis* complex (MTC) is a group of genetically similar subspecies responsible for tuberculosis in mammalian hosts (Riojas et al., 2018; Lipworth et al., 2019). Since the divergence of the MTC from the smooth tubercle bacilli (Supply et al., 2013), there is no evidence of gene acquisition by MTC members, and as such, the genomic architecture of MTC organisms is marked by reductive evolution (Mostowy and Behr, 2005). Despite gene deletion and accumulation of loss-of-function mutations, MTC members with more derivative genomes, such as *M. bovis*, are fully capable of productive infection and transmissible disease in a large number of mammalian hosts. Indeed, *M. bovis* has the broadest host-range of any MTC member (Morris et al., 1994; O’Reilly and Daborn, 1995): deer, badgers, and opossums have been reported as maintenance hosts (Brites et al., 2018), as well as infection in humans (Scott et al., 2016; Torres-Gonzalez et al., 2016).

It has been hypothesized that altered gene expression must underlie the capacity of organisms like *M. bovis* to adapt to new host niches, possibly through mutations in negative regulators (Garnier et al., 2003). Consistent with such a model, we previously documented that genes regulated by Sigma factor K (SigK) are constitutively expressed in *M. bovis* due to mutations in the putative anti-Sigma factor K, known as the Regulator of SigK (RskA) (Said-Salim et al., 2006). This was also suspected to be the case for *M. orygis* (formerly known as the Oryx bacillus) (Supply et al., 2013). We have shown that SigK controls the expression of eleven genes, including *mpt70* and *mpt83*, coding for homologous antigenic proteins MPT70 and MPT83 (Said-Salim et al., 2006), respectively. In addition, it has been reported that the expression of *katG* is influenced by SigK (Sklar et al., 2010).

Sigma factors initiate transcription of targeted genes by directing RNA polymerase to specific gene promoters. Based on phylogenetic relatedness, the σ70 family has been divided into several groups (Gruber and Gross, 2003), with those controlling some aspect of the cell surface or transport called ExtraCytoplasmic Function (ECF) Sigma factors (Lonetto et al., 1994). In general, an ECF is co-transcribed with its cognate anti-Sigma factor, which serves as a negative regulator by preventing the interaction between the ECF and the RNA polymerase until the appearance of a specific signal that allows its release (Helmann, 2002). *M. tuberculosis* has 13 σ factors, of which ten correspond to ECF σ factors (Manganelli, 2014), SigK being one of them.

The anti-ECF Sigma factor is often a membrane protein that transmits signals across the membrane to the cytoplasmic Sigma factor. Direct sensing of specific stimuli can either be done by the ECF, or by alternative proteins working in concert with the ECF. Several signaling mechanisms for activation of ECF Sigma factors have been described. An example is the FecA/TonB/FecR/FecI model of *Escherichia coli*, in which FecA initiates the signaling cascade by binding environmental metals which induces structural changes in FecA. With its partner TonB, FecA can interact with the anti-Sigma factor FecR, leading to the release of an active Sigma factor FecI that binds the RNA polymerase (Brooks and Buchanan, 2007). Another example is the SigE model of *E. coli*. SigE activation occurs upon proteolysis of the anti-Sigma factor RseB/A at the membrane by DegS and RseP proteases. This event releases the part of anti-Sigma factor RseA coupled to the SigE into the cytoplasm, whereupon a cytoplasmic protease cleaves SigE from RseA to initiate transcription (Brooks and Buchanan, 2007). Finally, in the SigB/SigF model of *Bacillus subtilis*, a “partner-switching” mechanism has been invoked, involving the activity of kinases (Igoshin et al., 2007).

Consistent with other regulators of ECF, *rskA* is co-transcribed with *sigK*. RskA is a predicted transmembrane protein, whose cytoplasmic N-terminal portion directly interacts with SigK (Said-Salim et al., 2006), and has been shown to inhibit the activity of this Sigma factor in different mycobacterial species (Veyrier et al., 2008). In *M. tuberculosis*, RskA is regulated by sequential proteolysis via RIP1 at cytoplasmic level (Rv2869c) (Makinoshima and Glickman, 2005; Sklar et al., 2010), while the protease responsible for the first cleavage outside the bacteria remains unknown. In addition, it has been shown that the oxidative state of two cysteines (133 and 183) in SigK influences its interaction with RskA and, therefore the signaling (Malkhed et al., 2011; Shukla et al., 2014). Under oxidizing condition, the disulfide bond formed between them stabilizes the SigK-RskA complex, whereas, in the presence of a reducing agent, SigK is released (Shukla et al., 2014). However, while RskA has been mutated independently twice in MTC members − *M. bovis* (G107D, G184E) and *M. orygis* (X233S) (van Ingen et al., 2014) (Supplementary Figure S1A), in neither case did the mutations affect the cytosolic portion of RskA, where the direct interaction with SigK occurs. It should be noted that the SNPs in the DNA that produce these amino acid changes are the only differences between the *sigK-rskA* locus from *M. tuberculosis*, *M. bovis* and *M. orygis* (i.e., SigK is identical between the three subspecies). While cytosolic RskA mutations might result in an impaired SigK inhibition through defective interaction, the molecular basis by which these extracytosolic mutations could change the expression of the regulon remains unclear (Said-Salim et al., 2006).

In this study, we used natural and artificial mutations in *rskA* (Supplementary Figure S1B) to improve our knowledge of the molecular mechanism of SigK regulation, revealing an unexpected activating role of RskA. Our findings indicate that RskA is a dual function activator-inhibitor, whereby extracytosolic RskA mutations cause increased SigK activity. In addition, we were able to reproduce these results using a SigK-RskA pair from the Gram-negative bacterium, *Yersinia enterocolitica* suggesting that this dual function may be widespread in the SigK family.

## Results

### Dominant Effect of Naturally Mutated Versions of RskA

We made a first observation that challenged the simple model of RskA as a pure inhibitor of SigK. Previously, we observed that wild-type RskA from *M. tuberculosis* partially suppressed the expression of the SigK regulon in *M. bovis* BCG Russia, but only when *rskA* was expressed under control of the highly active *hsp60* promoter (Said-Salim et al., 2006). Surprisingly, using the same construct, we were unable to suppress SigK activity in *M. orygis* or virulent *M. bovis* (data not shown). Under the simple inhibition model, it was expected that the absence of a functional RskA would liberate SigK to direct transcription of SigK-regulated genes, so the introduction of wild-type RskA would restore this defect. The difficulties encountered in these complementation studies stimulated an alternative hypothesis, in which mutated versions of RskA (G107D, G184E, or X233S) may be dominant over RskA*_*tuberculosis*_*.

**FIGURE 1:**
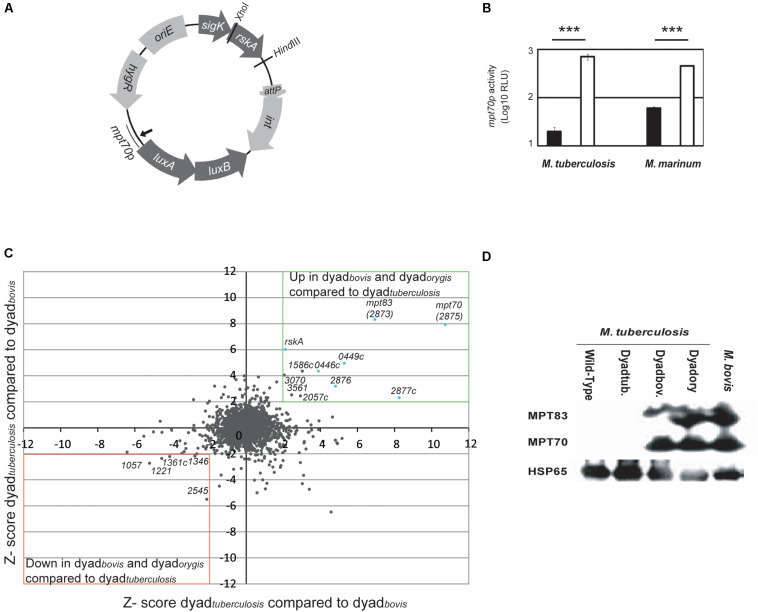
Dominant effect of mutated RskA on wild-type RskA. **(A)** Schematic representation of the plasmid backbone used in this study. **(B)** Luciferase expression of *M. tuberculosis* (H37Ra) and *M. marinum* harboring pMV::DyadRv-Lx70 (black) or pMV::Dyadbovis-Lx70 (white) which expressed, respectively, the *sigK-rskA* dyad from *M. tuberculosis* and *M. bovis*. Error bars represent standard deviations. Mutant forms of *rskA* result in over-expression of the SigK regulated genes, even in the presence of the wild-type *rskA*. Each bar represents the mean measurement for three independent clones (***p < 0.001). This representative data has been replicated at least twice. **(C)** Comparison of the activity of dyad*_*bovis*_* and dyad*_*orygis*_* with dayd*_*tuberculosis*_* assessed by microarray. Z-scores for *M. tuberculosis* H37Rv dayd*_*tuberculosis*_* (with pMV-Hyg::Dyad) compared with Dyad*_*bovis*_*. (pMV::Dyadbovis) (*y* axis, representing average of two arrays) have been plotted against Z-scores obtained when *M. tuberculosis* H37Rv dayd*_*tuberculosis*_* (with pMV-Hyg::Dyad) has been compared with Dyad*_*orygis*_*. (pMV::Dyadorygis) (*x*-axis, representing the average of two arrays). Genes whose expression is up-regulated in both cases are for the majority part of the *sigK* or *mpt70* locus (light blue). **(D)** Expression of MPT70, MPT83 and HSP65 (Loading control) measured by Western blot for *M. tuberculosis* H37Rv in native state (lane 1), *M. tuberculosis* after introduction of different *sigK-rskA* dyads (lanes 2-4) and *M. bovis* (lane 5). Dyadtub. indicates pMV-Hyg::Dyad, Dyadbov. indicates pMV::Dyadbovis and Dyadory indicates pMV::DyadOryx. This representative data has been replicated twice.

To test this possibility, we generated a plasmid harboring RskA variants along with luciferase fused to the promoter of *mpt70* (*mpt70*p) (Figure 1A). To approximate normal transcriptional conditions, the plasmid (pMV306::hyg) was integrated into the chromosome and the genes were under the control of a putative promoter located just before *sigK*, referred to as *sigKp* (Veyrier et al., 2008). The SigK activity reporter system was constituted by the *luxAB* genes from *Vibrio harveyi* controlled by *mpt70*p from *M. tuberculosis*, which we previously demonstrated is regulated only by SigK *in vitro* (Veyrier et al., 2008). These constructs were introduced in *M. tuberculosis* wild-type and *M. marinum*. Rather than including *rskA* alone, we used gene dyads (*sigK-rskA*) to assure that the ratio SigK/RskA is not modified. The luminescence produced by these bacteria is presented in Figure 1B. In *M. tuberculosis*, introduction of dyad*_*bovis*_* strongly increased *mpt70* promoter activity in the presence of wild-type RskA. The same results were observed in *M. marinum* which also possesses an inducible SigK regulon (Veyrier et al., 2008). To confirm that these results were not biased due to a problem in the plasmid, the *M. tuberculosis* genomic promoter response was directly evaluated using microarrays (Figure 1C) and indirectly by measuring the expression of MPT70 and MPT83 by immunoblot (Figure 1D). Our results show that the introduction of the dyad*_*tuberculosis*_* into *M. tuberculosis* did not affect the expression of MPT70 and MPT83. In contrast, the introduction of both dyad*_*bovis*_* and dyad*_*orygis*_* increased production of these proteins to a similar level than that observed in *M. bovis*. Taken together, these findings support the hypothesis that the mutated versions are dominant on the wild-type RskA.

### *M. bovis* and *M. orygis* RskA Variants Activate SigK

To explain the results seen with RskA*_*bovis*_* and RskA*_*orygis*_*, we hypothesized that (1) RskA can act as an activator of SigK, and (2) mutated versions of RskA from *M. bovis* and *M. orygis* are not merely non-functional – rather, these mutations result in SigK remaining in an activated state. To formally test the effect of these different natural mutants of RskA in a standardized and easy-to-use assay, we chose a mycobacterial species that lacks the *sigK-rskA* dyad, namely *M. smegmatis*. Into this organism, we introduced the plasmids containing both the *sigK-rskA* dyad regulatory core and a SigK-regulated reporter system (see Figure 1A). Moreover, we anticipated that this heterologous assay would be feasible because the SigK-binding sites are highly conserved between members of *Actinobacteria*, and because orthologues of SigK from different species can recognize the site in the *M. tuberculosis mpt70* promoter (Veyrier et al., 2008).

**FIGURE 2:**
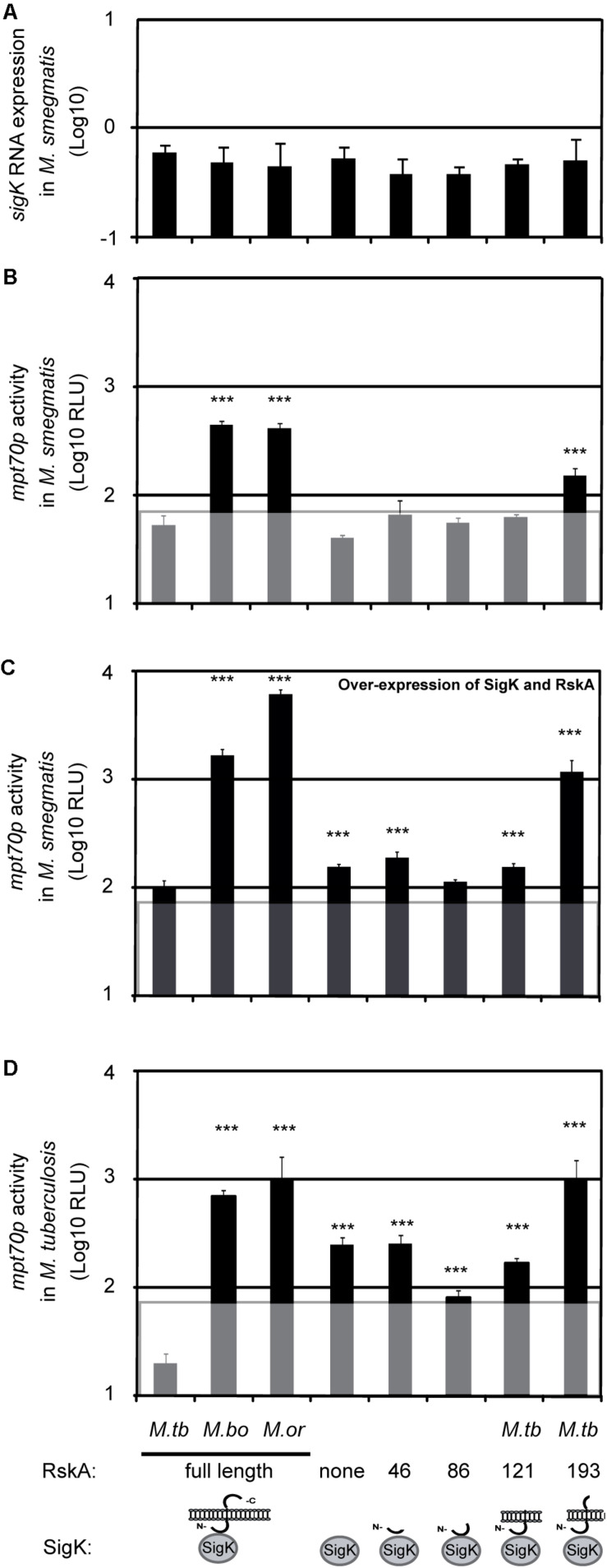
The anti-Sigma factor K (RskA) from *M. tuberculosis* is an activator and an inhibitor of SigK. **(A)** Relative expression of *sigK*, measured by RT-qPCR normalized with *sigA*, in *M. smegmatis* harboring the indicated constructs. Each bar represents the mean measurement for three independent clones. **(B–D)** Effects of RskA truncation or mutation on SigK activity, measured using luciferase under control of the promoter of *mpt70*. **(B)** Experiment conducted in the absence of a wild-type RskA (*M. smegmatis*). **(C)** Experiment conducted in the absence of a wild-type RskA (*M. smegmatis*) with over-expression of SigK and RskA and **(D)** Experiment conducted in the presence of a wild-type RskA (*M. tuberculosis* H37Ra). Error bars represent standard deviations for measurement of three independent clones. The background noise (line) was set at 1.8, based on replicate measurements of *M. smegmatis* expressing the luciferase under the promoter of *mpt70* but without *sigK* and *rskA* (data not shown). *M.tb* indicates *M. tuberculosis*, *M. bo* indicates *M. bovis* and *M.or* indicates *M. orygis*. Depending on the RskA forms, variable degrees of SigK modulation were observed. This representative data has been replicated twice (***p < 0.001 compared with dyad*_*tuberculosis*_*).

To validate the assay, we first tested it for already-documented effects of RskA mutations, by introducing plasmids coding for SigK along with different versions of RskA (from *M. tuberculosis*, *M. bovis*, or *M. orygis*). As expected, the dyad*_*bovis*_* and the dyad*_*orygis*_* resulted in ∼6-times more luciferase expression than the dyad*_*tuberculosis*_* (Figure 2B data point 1, 2, and 3). Next, we measured the activity of SigK alone by removing RskA from the plasmid previously described (pMV::dyadbovis-Lx70) and measuring luciferase activity in *M. smegmatis* (Figure 2B, data point 4). In the presence of full-length RskA*_*bovis*_* (data point 2) or RskA*_*orygis*_* (data point 3), we observed RLU superior than that observed when RskA was absent (i.e., SigK alone, data point 4). Therefore, in addition to the inhibitor function of RskA (documented previously, Said-Salim et al., 2006), it can be an activator of SigK activity. As a control, we have measured *sigK* RNA expression using RT-qPCR in *M. smegmatis* harboring the different plasmids to test for construction-mediated changes in *sigK* expression which could influence SigK activity. Control measurements showed no significant differences in *sigK* expression between constructs (Figure 2A). To test whether removing the RskA*_*bovis*_* or RskA*_*orygis*_* had similar effects in *M. tuberculosis*, we introduced the same constructs into the H37Ra strain of *M. tuberculosis*. As shown in Figure 2D, the presence of RskA*_*bovis*_* (data point 2) or RskA*_*orygis*_* (data point 3) resulted in superior activity than the one observed when SigK is alone (data point 4). Of note, in *M. smegmatis*, the plasmid versions of the dyads are under control of *sigK*p, which is not SigK regulated, enabling us to quantify the expression of our construct reliably. Besides, we reproduced this effect by changing *sigK*p by the overexpressing promoter *hsp60*p (Figure 2C). However, in *M. tuberculosis*, the genomic version of the gene is autoregulated via Rv0449cp (Charlet et al., 2005; Said-Salim et al., 2006; Veyrier et al., 2008), therefore we have not quantified *sigK* expression by RT-qPCR.

### Artificial Truncation Can Also Block RskA Into Its SigK-Activating Conformation

In previous studies with FecI-FecR, a similar effect of FecI-activation by FecR was observed using artificial truncation of the anti-Sigma factor (Ochs et al., 1995, 1996). To further explore the Sigma/anti-Sigma partnership, beyond the natural mutation, we constructed different versions of the plasmid coding for SigK along with truncated versions of RskA. Schematic representations of the different RskA truncations are presented at the bottom of Figure 2 (data points 5-8) and in Supplementary Figure S1. Again, *sigK* expression by RT-qPCR was similar across constructs, enabling us to infer that any differences observed were due to the RskA variants (Figure 2A). Interestingly, we could observe that the removal of a specific fragment of the C-terminal part of RskA*_*tuberculosis*_* (194 to 232, data point 8) led to the same phenotype observed with RskA*_*bovis*_* or RskA*_*orygis*_* (data points 2 and 3) whereas further truncation (122 to 232, data point 7 or 87 to 232, data point 6) did not. This suggests that the extra cytoplasmic tail (194 to 232) is controlling the dual activity of RskA and removing it has the same effect as mutation (G107D, G184E, or X233S).

### Artificial Truncation Can Also Block RskA Into Its SigK-Activating Conformation in Gram-Negative Bacteria

**FIGURE 3:**
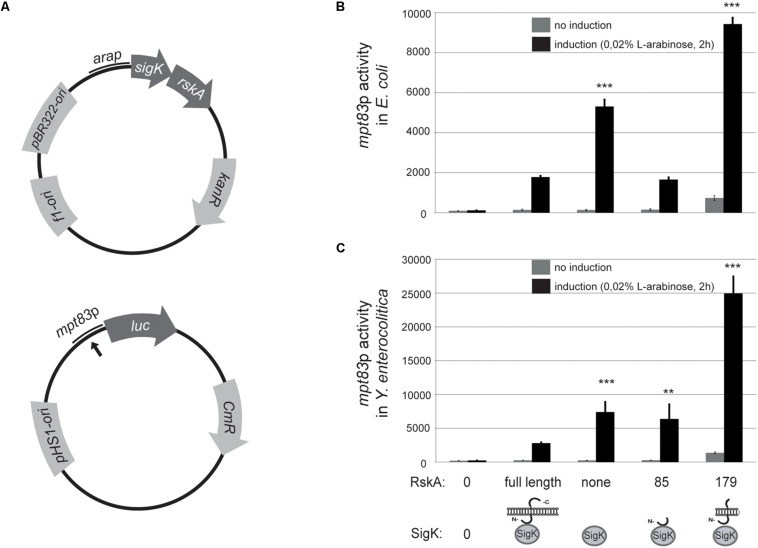
**(A)** The anti-Sigma factor K (RskA) from *Y. enterocolitica* is an activator and an inhibitor of SigK. **(A)** Schematic representation of the plasmid backbones used in this study. Bold black arrow represents predicted SigK binding site. Truncated versions of RskA from *Y. enterocolitica* and its effect on SigK activity measured by luciferase expression under control of *mpt83* promoter in *E. coli* **(B)**, and *Y. enterocolitica* **(C)**. Error bars represent standard deviations of three independent experiments. Non-induction and induction with L-arabinose are shown in both cases (**p < 0.01, ***p < 0.001 compared with SigK and full-length RskA).

To explore whether the findings are specific to *Mycobacteria*, we tested this model in a genetically different bacteria that harbor *mpt83-sigK-rskA* minimal cassette (Veyrier et al., 2008) namely *Y. enterocolitica*. To prevent confusion, MPT83 and MPT70 are paralogous genes in *M. tuberculosis* that arose from a duplication. We have already shown that MPT83 (with a lipid anchor) is the ancestral version (Veyrier et al., 2008). Therefore, we called the gene *mpt83* (Veyrier et al., 2008) and have already shown that a SigK-binding site is present upstream of the gene. A similar strategy was taken with heterologous expression in *E. coli* (another proteobacteria but that do not contain SigK nor RskA) and *Y. enterocolitica* wild-type. We constructed a plasmid (pST76-K) that harbors *mpt83*p from *Y. enterocolitica* fused to the firefly luciferase (Figure 3A). Different versions of *sigK* and *rskA* were cloned into pBAD and expressed with the arabinose inducible promoter (Figure 3A). We expressed truncated versions of RskA*_*enterocolitica*_* (1 to 179) or (1 to 85), which correspond to the same truncations studied for RskA*_*tuberculosis*_*: (1 to 193) and (1 to 86), respectively (see the alignment in Supplementary Figure S1). Again, whether expressed in *E. coli* (Figure 3B) or *Y. enterocolitica* (Figure 3C), RskA had the same effect on SigK: no activity with the full-length RskA or with 1 to 85 version (inhibition); some activity with SigK alone (low on); full activation with RskA from 1 to 179 (high on). These data suggest that the mechanism of SigK activation by RskA is conserved across these genera.

### RskA Does Not Stabilize SigK

**FIGURE 4:**
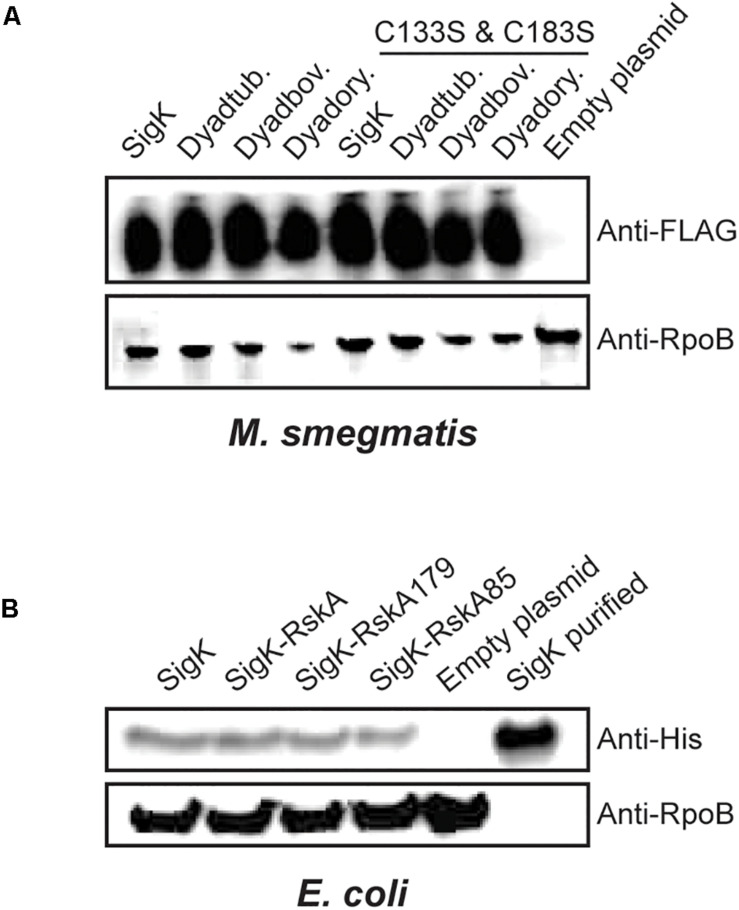
SigK activity occurs without degradation of SigK protein. **(A)** Expression of SigK, with a FLAG-tag linked to the N-terminus, was detected by immunoblot using monoclonal FLAG-tag antibodies in *M. smegmatis* wild-type, or expressing SigK alone; Dyad*_*tuberculosis*_*, Dyad*_*bovis*_* and Dyad*_*orygis*_* and the SigK-cysteine-less versions. **(B)** Similarly, expression of SigK with a His-tag linked to the N-terminus, was detected by immunoblot using monoclonal His-tag antibodies in *E. coli* expressing SigK alone; SigK-RskA; SigK-RskA179 or SigK-RskA85. Purified SigK was used as a positive control and *E. coli* harboring the empty plasmid as a negative control. RpoB was used as a loading control in both cases using an anti-RpoB antibody that react with both species. This representative data has been replicated twice.

Based on prior studies on SigK activation, we hypothesized that RskA could undergo sequential proteolysis and that a small remaining fragment of RskA would stabilize SigK. Proteolysis of Sigma factors has been demonstrated for SigT in *Streptomyces coelicolor* (Mao et al., 2013). Therefore, we tested if SigK is stable with the different versions of RskA in both mycobacteria and proteobacteria. To evaluate this possibility, we first added a FLAG-tag at the N-terminal part of SigK*_*tuberculosis*_* and showed that the activity was unaffected by the tag (Supplementary Figure S2A). Next, we measured SigK levels by immunoblot against FLAG-tag in *M. smegmatis*, revealing that SigK activity, which varies widely according to the RskA, was uncoupled from the amount of SigK detected (Figure 4A). We performed the same in proteobacteria by expressing the different versions of the dyad with a His-tag in the N-terminal part of SigK*_*enterocolitica*_* in *E. coli* using pET-28a plasmid. Again, the tag did not alter the activity of SigK (low on, on and high on) (Supplementary Figure S2B) and we still did not observe changes in SigK stability correlated with its activity (Figure 4B).

### SigK-Cysteines Are Important for Its Activity

**FIGURE 5:**
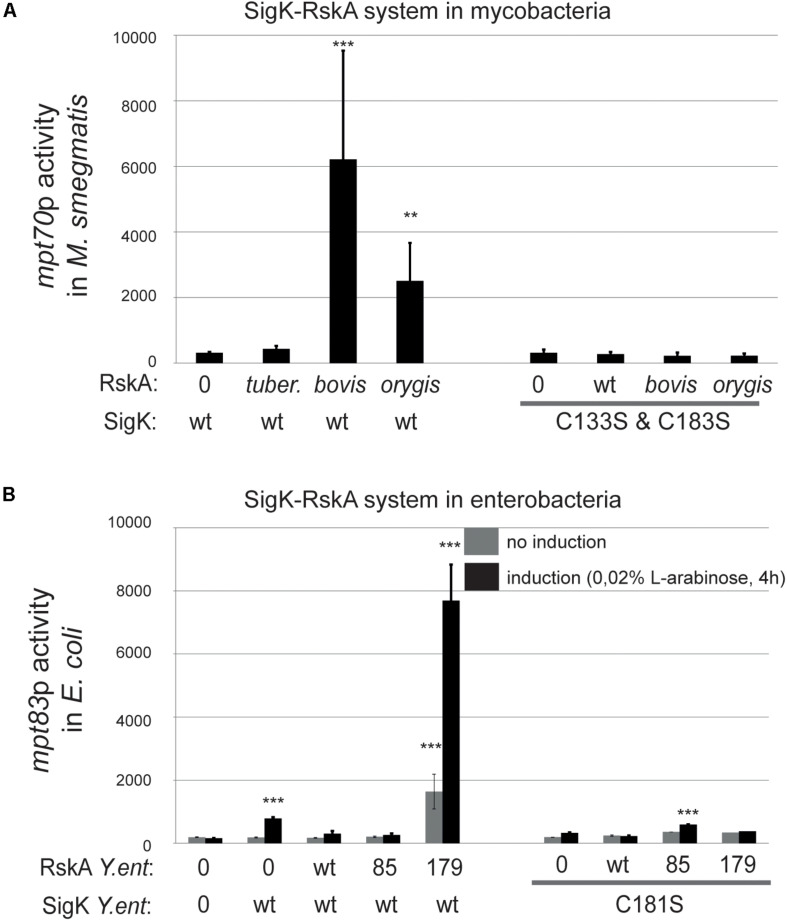
SigK activity requires two conserved cysteines carried by SigK. Effect of cysteines vs. serines on SigK activity were measured by luciferase under the control of *mpt70p* in *M. smegmatis* **(A)** and *mpt83p* in *E. coli* **(B)**. Error bars represent standard deviations of three independent experiments with 2 different clones each time. **p < 0.01, ***p < 0.001.

Cysteines (133 and 183) in SigK from *M. tuberculosis* in their oxidized state have been shown to influence the RskA-SigK interaction, and therefore signaling (Malkhed et al., 2011; Shukla et al., 2014). We tested whether interfering with the interaction would affect activity by replacing these two cysteines with serines. As seen in Figure 5, activation was abrogated for both the Dyad*_*bovis*_* and Dyad*_*orygis*_* in *M. smegmatis* (Figure 5A) and in *M. tuberculosis* Δ*sigK* (Supplementary Figure S3). To verify that these SigK versions are all expressed and stable, we used a FLAG-tag fusion strategy as explained before and we measured SigK levels by immunoblot in *M. smegmatis*, revealing that SigK and cysteines-less SigK are expressed similarly (Figure 4A).

We performed a similar experiment in *E. coli* by expressing the different versions of RskA*_*enterocolitica*_*, again with SigK where cysteine 181 was replaced by a serine (Figure 5B). Without these cysteines, SigK activity is minimal and no activation by the truncated RskA is observed. This data supports a model where SigK requires the two cysteines to be fully activated by RskA.

### Bioinformatic Search for Anti-Sigma Factor Adaptation Toward Cysteines Absence in SigK

**FIGURE 6:**
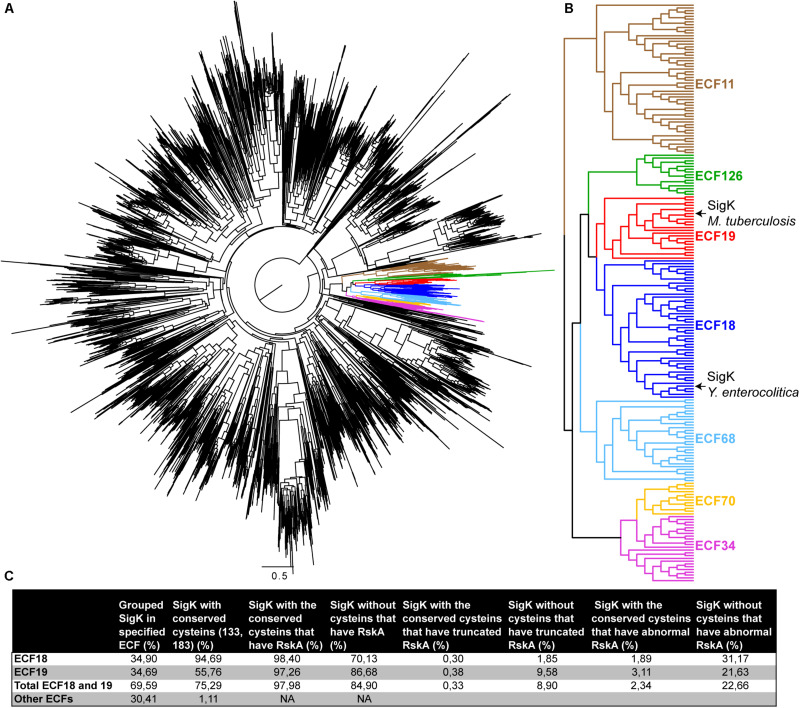
Species with SigK that naturally evolved without cysteines have adapted RskA **(A)** Phylogenetic tree of 3717 ECF sequences done as described in the Materials and Methods section. Specific ECF families are colored, as also shown in panel **(B)**. **(C)** Statistics on the SigK/RskA based on all representative sequences available and found in RefSeq, as described in the section “Materials and Methods”.

Several studies have grouped Sigma factors in different families (Staron et al., 2009; Pinto and Mascher, 2016). We present in Figure 6 a phylogeny of all the Sigma factors (Figure 6A), showing that SigK*_*tuberculosis*_* is part of the ECF18 and SigK*_*enterocolitica*_* is part of the neighboring ECF19 family (Figure 6B). To study these two families in more detail, we searched for SigK in all the representative genomes of the RefSeq database (11,072 sequences). We recovered 4157 SigK sequences distributed across 22 families (Supplementary Table S1). As expected, we found around 35% of ECF18 and 35% of ECF19. The other represented families are ECF01 (11%); ECF34 (8%); ECF11 (6%); and ECF70 (3%). Next, we aligned all the proteins and checked for cysteines 133 and 183 conservation. These cysteines are conserved in 94% in ECF18 and in 56% in ECF19, but less than 2% of other ECF families, suggesting that this may be a specific feature of these two families. To better characterize the partnership between SigK and RskA and the role of these cysteines, we searched for RskA homologous sequences by BLASTP with the requirement that the RskA be adjacent to the identified SigK, considering the polarity of the genes. In the event that no RskA was found, the adjacent gene was still extracted. We next analyzed the obvious differences in RskA between loci that contain SigK (ECF18 and ECF19) with or without the two cysteines (Figure 6C). We first verified the presence of RskA. The absence of RskA was defined by a complete absence of anti-Sigma factor or an anti-Sigma factor from a different family than RskA. In the group SigK with the 2 cysteines, 98% of them harbor a RskA whereas this proportion decrease to 85% in the group of SigK without cysteines (Figure 6C). In repeated instances, we detected species with a truncated RskA (with an arbitrary threshold of <150AA). In the group SigK with the 2 cysteines, 0.3% of them harbor a truncated RskA; this proportion increased to 9% in the group of SigK without cysteines (Figure 6C). Overall, 23% of SigK without the 2 cysteines harbor abnormal RskA (as compared to 2% in SigK with cysteines) (Figure 6C). This firmly suggests a strong ongoing adaptation of this Sigma factor/anti-Sigma factor partnership for several species that have members from these two families of ECF18 and ECF19 (as we first detected in *M. bovis* and *M. orygis*) and particularly for those that do not have conserved these two cysteines.

### SigK Without Cysteines, Found in *Nocardia spp.*, Are Functional Without RskA Activation

**FIGURE 7:**
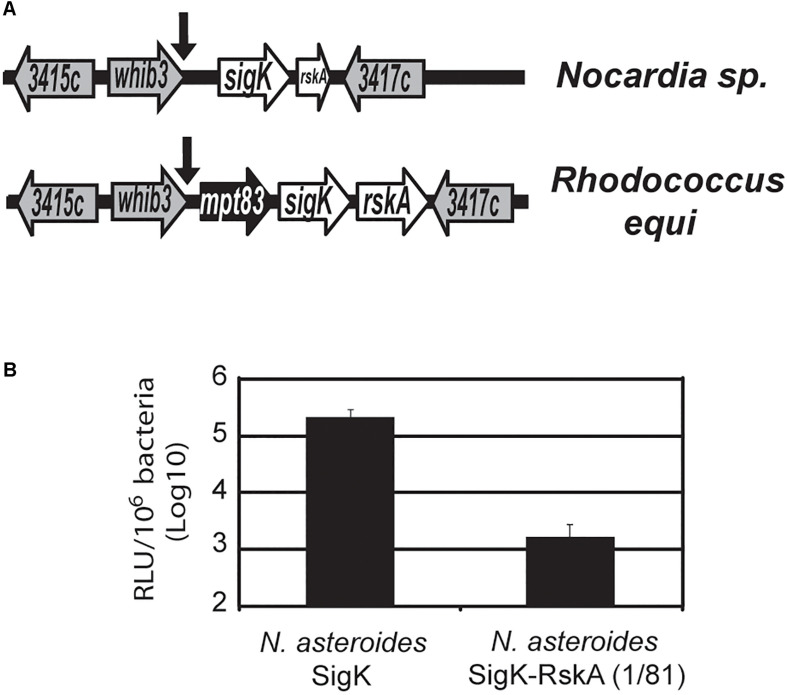
SigK that naturally evolved without cysteine is functional. **(A)** Schematic representation of *sigK* locus in *Nocardia* sp. and *Rhodococcus equi*. In *Nocardia*, the *rskA* gene encodes a truncated protein (1 to 81) and *mpt83*, a core member of the conserved *mpt83-sigK-rskA* cassette (Veyrier et al., 2008), has been deleted. The arrows indicate SigK binding sites and genes are named based on their *M. tuberculosis* H37Rv number. **(B)** Luciferase expression of *M. smegmatis* with plasmid containing *sigK*_*nocardia*_ or *sigK*_*nocardia*_ and *rskA*_*nocardia*_ (1 to 81). Error bars represent standard deviations. In the absence of the first 81 amino acids of RskA, there is a strong activation of *mpt70p*. Each bar represents the mean measurement for three independent clones. This representative data has been replicated at least twice.

When we mutated the cysteines, SigK activity was minimal and RskA activation was not observed. But several species harbor SigK without cysteines. To understand these natural cysteine-less SigK, we again used an evolutionary method, looking for natural mutants, in this case outside of the *M. tuberculosis* complex. We previously described a cassette consisting of *mpt83-sigK-rskA* (Veyrier et al., 2008) with a SigK binding site that is conserved even in Gram-negative bacteria (e.g., *Yersinia* sp.). The genera *Nocardia* sp., *Rhodococcus* sp. and *Mycobacterium* sp. are part of the same suborder *Corynebacterineae*. While both *Rhodococcus* sp. and *Nocardia farcinica* have a full-length *sigK*, in *N. farcinica* the *rskA* is truncated (coding for a protein of 81 amino acids) and *mpt83* is absent (Figure 7A). To test whether the premature stop codon was a general feature of *Nocardia* sp., we sequenced this region in *Nocardia asteroides* 42007, *N. nova* 90960, and *N. abscessus* 91107. In each of these species, we detected the same stop-codon mutation (and the absence of *mpt83*). Interestingly, the cysteines are absent in these species (whereas they are conserved in all the other mentioned species harboring a SigK and a non-truncated RskA)

To test the potential consequence of this form of RskA and SigK, we introduced the *sigK-rskA* dyad from *Nocardia asteroides* into the plasmid containing *luxAB* under the control of *mpt70p* (Supplementary Figure S1). As a control, we engineered a RskA-less plasmid, by introducing a stop codon in the *rskA* gene after *sigK* stop codon. After electroporation into *M. smegmatis*, we observed that removing the remaining part of RskA results in a ∼2 log increased in luciferase activity compared to SigK alone (Figure 7B). These findings indicate that SigK alone is highly active and the presence of the first 81 amino acids of RskA*_*asteroides*_* inhibited SigK*_*asteroides*_*. Of note, we have previously shown that amino acids 1 to 92 from RskA are sufficient to bind SigK (Said-Salim et al., 2006) and have demonstrated that amino acids 1 to 81 of RskA inhibit SigK (Figures 2, 3). These data support a model in which RskA, like any other anti-Sigma factors, acts as an inhibitor of its cognate Sigma factor, through its N-terminal portion. In the specific case of *Nocardia* sp., in the absence of RskA we observed a strong induction of *mpt70p*, to a much greater degree than was observed in the case of SigK*_*tuberculosis*_* alone and similar to what was observed with dyad*_*bovis*_* or dyad*_*orygis*_*. This suggests that SigK*_*asteroides*_* is able to strongly act on *mpt70p* promoter without the need for a RskA-mediated switching event. A unique aspect of this particular Sigma factor is the absence of the two cysteines which are conserved in other SigK sequences from ECF18 and ECF19. It is important to note that with recent sequencing efforts and increased *Nocardia* genome sequences available for comparison, certain strain of *Nocardia* with the archetype of SigK and RskA can be observed (Supplementary Table S1): for example, *Nocardia* sp. 348MFTsu5.1 harbors a full-length RskA (H290_RS0112295) and a SigK with the two cysteines (H290_RS0112290), indicating again that the these two cysteines in SigK are found concomitantly with the ancestral full-length RskA.

## Discussion

The detailed study of Sigma factor activation offers insights into the processes used by bacteria to respond to the stresses they encounter during their lifecycle. In this study, we have explored natural and engineered mutations in RskA (the anti-Sigma factor of SigK) to investigate the signal transduction process of the SigK regulon in Actinobacteria, and also Gram-negative bacteria. In *Mycobacteria*, using a number of different approaches, our investigations demonstrate that increased SigK activity on the SigK-regulated promoter does not happen simply because of loss-of-function mutations in the respective forms of RskA in *M. bovis* and *M. orygis*. Rather, these two independently mutated forms of RskA, whether introduced into *M. smegmatis* or *M. tuberculosis*, actively turn on SigK, even in the presence of wild-type RskA. We also demonstrated that the same effect could be obtained by removing a part of the C-terminal part of RskA in both *Mycobacteria* and *Enterobacterales*. Based on our results, we infer that SigK has at least three degrees of activity: (i) “Off” when inhibited by RskA, (ii) “Low-On” when alone, and (iii) “High-On” when activated by RskA. The first two states are generally used to identify a protein as an anti-Sigma factor, unlike the third condition (activation) which is rarely tested. One exception is the anti-Sigma factor FecR which has been shown by different groups to activate the cytoplasmic Sigma factor FecI in different bacteria (Ochs et al., 1995; Llamas et al., 2009; Llamas et al., 2009). Interestingly, the description of FecI activation by FecR would have been impossible without mutagenic studies of FecR (Ochs et al., 1995, 1996), in the same way that the activating properties of RskA would not have been suspected if not for the natural mutants in *M. bovis* and *M. orygis*. Importantly, there are clear differences between the FecR model and our data on RskA. The key distinction is that the FecI-modulating domain of FecR (N-term 1 to 85) is an activator when separated from the rest of the protein (Stiefel et al., 2001). In RskA, the equivalent domain of RskA is an inhibitor on its own. Based on this, we predict that the activating function of other anti-Sigma factors could be underappreciated.

As the signal that activates the signaling cascade is still unknown (we have not found any condition that is able to activate *mpt70*p, except contact with macrophage, Veyrier et al., 2008), we were unable to test the precise events that initiate the cascade. In *M. tuberculosis*, it has been shown that RskA is hydrolyzed by an intramembrane hydrolase Rip1(Rv2869c) that belongs to site-2-protease (S2P) (Sklar et al., 2010). The S1P protease of RskA in *M. tuberculosis* remains unknown. Interestingly, a recent study showed that some anti-Sigma factors have an auto-cleavage between the periplasmic Gly191 and Thr192 residues (Bastiaansen et al., 2015). These residues are present in RskA and this motif is the one mutated (G193E) in *M. bovis*. Furthermore, we have shown that the C-terminal part of RskA has an inhibitory effect on RskA activating function. Our findings, along with observations from other organisms, suggest that activation of SigK by RskA could start with a modification of C-terminal portion of RskA. Therefore, a possible hypothesis would be that a sequential proteolysis of RskA could result in a small fragment of RskA that promotes SigK activity, as has been proposed for FecIR and some anti-Sigma factors from *Pseudomonas* sp. (Otero-Asman et al., 2019), and alteration of the C-terminal part of RskA exacerbates this. Although the mechanism of cascade activation is not understood, our data report on the signal transmission mechanism between RskA and SigK. A recent study determined the crystal structure of SigK in complex with the cytoplasmic domain of RskA and revealed the presence of disulfide bridge involved in the stability of the partnership (Shukla et al., 2014). Our findings support and build upon these results. We have confirmed the importance of these cysteines in signal transduction of SigK in both *Mycobacteria* and *Enterobacterales*. One possible mechanism for SigK activation through RskA would be through the protection against proteases of SigK by RskA, as has been demonstrated in *Streptomyces coelicolor* (Mao et al., 2013). In our model, the inactivated SigK system of *M. tuberculosis* does not appear to occur through SigK instability. Finally, it could also be possible that SigK recognizes a signal through the cysteine bridge and that RskA is modulating this sensing via its interaction with SigK.

Interestingly, upon further characterization of the ECF18 and ECF19, we also shed light on the “recent” ongoing evolution of these family of Sigma factor/anti-Sigma factors in multiple instances. The co-occurrence of the cysteines with full-length RskA suggests a to-be-determined role of these cysteines in the switching On/Off of SigK by full-length RskA. We found that absence of the two cysteines is correlated with changes in the anti-Sigma factor (as compared to the RskA potential archetype). This could be the presence of another anti-Sigma from a different family than RskA but also, in numerous cases, mutations in RskA. For example, we found that the majority of *Nocardia spp.* have evolved a Sigma-factor without cysteine and a truncated RskA (around 80 amino acids). It is interesting to note that, unlike when we artificially mutated the cysteines in SigK, naturally evolved cysteine-less SigK are highly active suggesting of compensatory amino-acid changes. In addition, the little remnant of RskA, although often annotated as a pseudogene, is still “functional” and serves as an inhibitor of SigK. The species-specific evolution of this family is complexifying the study of the SigK/RskA partnership. In the light of these examples of species-specific evolution (*M. bovis, M. orygis, Nocardia spp.*) the study of the partnership between SigK and RskA could be complicated and imply some species-specific characteristics. Nevertheless, these species have all lost or altered the part of RskA that is supposed to sense the signal. Again, this may argue for the possibility that SigK would also sense a specific/alternative signal.

In conclusion, our results emphasize a broader and complex function for anti-Sigma factors as modulators (inhibitor/activator) of the Sigma factor, rather than solely acting as inhibitors. Importantly, the nature of the signal which initiates this cascade is still unknown. Nevertheless, this knowledge can serve to engineer differential expression of the SigK regulon in the same bacterial background and generate tools to decipher in detail the transcriptional activation and the function of this regulon. A similar strategy, using natural and engineered anti-Sigma factor mutations, represents an attractive approach to control the activity of specific Sigma factors, which serves to understand the function of their regulons.

## Materials and Methods

### Bacterial Strains, Culture Conditions, and Genomic DNA

*Mycobacterium orygis* 51145 (Mostowy et al., 2005; Said-Salim et al., 2006), *M. bovis* 68799 (gifts from Louise Thibert), *M. tuberculosis* H37Rv, *M. tuberculosis* H37Ra, *M. marinum* ATCC BAA-535, and *M. smegmatis* str. MC2 155 were grown at 37°C (except for *M. marinum*; 30°C) under rotating conditions, in Middlebrook 7H9 medium (Difco Laboratories, Detroit, MI, United States) containing 0.05% Tween 80 (Sigma-Aldrich, St. Louis, MO, United States) and 10% Albumin-Dextrose-Catalase (Becton Dickinson and co., Sparks, MD, United States). Middlebrook 7H10 media was used as the solid medium supplemented with 10% Oleic acid-Albumin-Dextrose-Catalase (Becton Dickinson and co., Sparks, MD, United States). *Yersinia enterocolitica* DSMZ 23249 and *E. coli* were grown in Luria-Bertani Medium (Difco) at 30°C. As required, antibiotics were added as follows: 50 μg.ml^–1^ kanamycin for *E. coli and Y. enterocolitica* and 25 μg.ml^–1^ for *Mycobacteria*, 100 μg.ml^–1^ hygromycin for *E. coli* and 50 μg.ml^–1^ for *Mycobacteria*, 25 μg.ml^–1^ chloramphenicol for *E. coli and Y. enterocolitica*, and 100 μg.ml^–1^ ampicillin for *E. coli*. All the strains used in this study are summarized in Table 1.

**TABLE 1:** Strains and plasmids used in this study.

Strains or plasmids	Relevant genotype description	Reference
**Strains**		
*E. coli* DH5α	Host for cloning	NEB
*E. coli* TOP10	Host for cloning	Invitrogen
*E. coli* BL21 (DE3) pLysS	Host for expression vector	NEB
*M. smegmatis* mc^2^ 155	Lab strain	
M. tuberculosis H37Ra	Lab strain	
*M. tuberculosis* H37Rv	Human isolate	
*M. bovis* 68799	Bovine isolate	
*M. orygis* 51145	Antilope isolate	[44]
*Y. enterocolitica* DSMZ 23249	Lab strain	
**Plasmids**		
pMV306	Integrase, Hyg^r^	Said-Salim et al., 2006
pMV306::DyadM.tb	Integrase, *sigK* and *rskA* of *M. tuberculosis*, Hyg^r^	Said-Salim et al., 2006
pMV306::DyadM.bo	Integrase, Hyg^r^, *sigK* of *M. tuberculosis* and *rskA* of *M. bovis*	This study
pMV306::DyadM.or	Integrase, Hyg^r^, *sigK* of *M. tuberculosis* and *rskA* of *M. orygis*	This study
pLx-MPT70	*luxAB* genes under *mpt70* promoter, replication origin *E.coli*, Hyg^r^	Veyrier et al., 2008
pMV306-Lx	Integrase, *luxAB* genes under *mpt70* promoter, Hyg^r^	This study
pOE-Lx	Integrase, *luxAB* under *mpt70* promoter, *hsp60* promoter, Hyg^r^	This study
pET28b+	Replication origin *E.coli*, T7 promoter, Km^r^	Novagen
pMV306-Lx::SigKalone	*sigK* of *M. tuberculosis*	This study
pMV306-Lx::DyadM.tb	*sigK* and *rskA* of *M. tuberculosis*	This study
pMV306-Lx::DyadM.bo	*sigK* of *M. tuberculosis* and *rskA* of *M. bovis*	This study
pMV306-Lx::DyadM.or	*sigK* of *M. tuberculosis* and *rskA* of *M. orygis*	This study
pMV306-Lx::Dyad1/193M.tb	*sigK* and *rskA* 1/193 of *M. tuberculosis*	This study
pMV306-Lx::Dyad1/193M.bo	*sigK* of *M. tuberculosis* and *rskA* 1/193 of *M. bovis*	This study
pMV306-Lx::Dyad1/86M.tb	*sigK* and *rskA* 1/86 of *M. tuberculosis*	This study
pOE-Lx::SigKalone	*sigK* of *M. tuberculosis*	This study
pOE-Lx::DyadM.tb	*sigK* and *rskA* of *M. tuberculosis*	This study
pOE-Lx::DyadM.bo	*sigK* of *M. tuberculosis* and *rskA* of *M. bovis*	This study
pOE-Lx::DyadM.or	*sigK* of *M. tuberculosis* and *rskA* of *M. orygis*	This study
pOE-Lx::SigKaloneSer	*sigK* C133S − C183S of *M. tuberculosis*	This study
pOE-Lx::DyadSerM. tb	*sigK* C133S − C183S and *rskA* of *M. tuberculosis*	This study
pOE-Lx::DyadSerM.bo	*sigK* C133S − C183S of *M. tuberculosis* and *rskA* of *M. bovis*	This study
pOE-Lx::DyadSerM.or	*sigK* C133S − C183S of *M. tuberculosis* and *rskA* of *M. orygis*	This study
pOE-Lx::SigKaloneFlag	*sigK* FLAG-tag in N-terminal of *M. tuberculosis*	This study
pOE-Lx::DyadM.tbFlag	*sigK* FLAG-tag in N-terminal and *rskA* of *M. tuberculosis*	This study
pOE-Lx::DyadM.boFlag	*sigK* FLAG-tag in N-terminal of *M. tuberculosis* and *rskA* of *M. bovis*	This study
pOE-Lx::DyadM.orFlag	*sigK* FLAG-tag in N-terminal of *M. tuberculosis* and *rskA* of *M. orygis*	This study
pOE-Lx::SigKaloneSerFlag	*sigK* C133S − C183S FLAG-tag in N-terminal of *M. tuberculosis*	This study
pOE-Lx::DyadSerM.tbFlag	*sigK* C133S − C183S FLAG-tag in N-terminal and *rskA* of *M. tuberculosis*	This study
pOE-Lx::DyadSerM.boFlag	*sigK* C133S − C183S FLAG-tag in N-terminal of *M. tuberculosis* and *rskA* of *M. bovis*	This study
pOE-Lx::DyadSerM.orFlag	*sigK* C133S − C183S FLAG-tag in N-terminal of *M. tuberculosis* and *rskA* of *M. orygis rskA* of *M. tuberculosi*	This study
pKO5luc	*luc* gene under *mpt83* promoter, Cm^r^	This study
pBAD-Km::SigK	*sigK* of *Y. enterocolitica*, Km^r^	This study
pBAD-Km::SigK-RskA	*sigK* and *rskA* of *Y. enterocolitica*, Km^r^	This study
pBAD-Km::SigK-RskA179	*sigK* and *rskA* 1/179 of *Y. enterocolitica*, Km^r^	This study
pBAD-Km::SigK-RskA85	*sigK* and *rskA* 1/85 of *Y. enterocolitica*, Km^r^	This study
pBAD-Km::SigKC181S	*sigK* C181S of *Y. enterocolitica*, Km^r^	This study
pBAD-Km::SigKC181S-RskA	*sigK* C181S and *rskA* of *Y. enterocolitica*, Km^r^	This study
pBAD-Km::SigKC181S-RskA179	*sigK* C181S and *rskA* 1/179 of *Y. enterocolitica*, Km^r^	This study
pBAD-Km::SigKC181S-RskA85	*sigK* C181S and *rskA* 1/85 of *Y. enterocolitica*, Km^r^	This study
pET28a::SigK	*sigK* of *Y. enterocolitica*, Km^r^, His-tag in *sigK* N-terminal	This study
pET28a::SigK-RskA	*sigK* and *rskA* of *Y. enterocolitica*, Km^r^, His-tag in *sigK* N-terminal	This study
pET28a::SigK-RskA179	*sigK* and *rskA* 1/179 of *Y. enterocolitica*, Km^r^, His-tag in *sigK* N-terminal	This study
pET28a::SigK-RskA85	*sigK* and *rskA* 1/85 of *Y. enterocolitica*, Km^r^, His-tag in *sigK* N-terminal	This study

### Mycobacteria Plasmids Construction

(1) Plasmid containing *sigK-rskA* full length: The construction of plasmids coding for *sigK-rskA* from *M. tuberculosis* (pMV306-Hyg::dyad) and *M. bovis* (pMV::dyadbovis) have been already described (Said-Salim et al., 2006; Veyrier et al., 2008). To construct pMV::dyadoryx a part of the *sigK-rskA* region has been amplified, from genomic DNA of *M. orygis*, using Rv0444OF and Rv0444OR primers (Table 2). This amplicon was ligated to pMV306-Hyg::dyad digested with XhoI and HindIII (Figure 1A). Secondly, the reporter system was added to these plasmids: pLx-MPT70 (Veyrier et al., 2008) was digested using XbaI-SphI to extract *luxAB* fused to *mpt70p*. This fragment was treated with T4 polymerase to generate blunt ends and ligated with pMV306-Hyg::dyad, pMV::dyadbovis or pMV::dyadoryx digested by BsrDI. Doing that, pMV::dyadRv-Lx70, pMV::dyadbovis-Lx70 and pMV::dyadoryx-Lx70 have been respectively, generated.

**TABLE 2:** Oligonucleotide primers used in this study.

Primers name	Sequence (5′→3′)
Rv0444OF	GTG**CTGCAG**GGTGCGGCCAACG
Rv0444OR	GGA**AAGCTT**GATAACGGCGACATC
RskA1/46R	CTC**AAGCTT**AGTCGTTGAAAGCCGC
RskA1/86R	GCC**AAGCTT**CAGCGTGATTGGCGC
RskA1/121R	GCT**AAGCTT**CGGTGGGGGGTGG
RskA1/193R	CCG**AAGCTT**AGGGCGTCACCGCC
RskA71/232F	CAT**CTCGAG**ACGGCCATCCTGGAT
NfRv3416F	GACGTCTGGGACTGGCAGATGC
NfRv3417R	CGCATGGTGCTCACCACCGAG
PpuMIStopF	GACTAGTAAGTGAGTAGGGC
PpuMIStopR	GTCGCCCTACTCACTTACTA
SigAMsF	CCAAGGGCTACAAGTTCTCG
SigAMsR	TGGATCTCCAGCACCTTCTC
SigKMunIF	GACCCAATTGACCATCACGGCGC
Rv0444RHindIII	ATAAAAGCTTCCGGCGTGTTCGTCGCGATGC
SigK133F	CGCCGGGTGACCGAGTCCCTCAAGGCGTTGACCGAC
SigK133R	GTCGGTCAACGCCTTGAGGGACTCGGTCACCCGGCG
SigK183R	GGGAAGCTTACTCGAGCAGCTCAAAATCG GTATGTTCAGTCATGAGCGCCGCTCTCCCAAC GCATCGCTTCGCTCGGCCGGCGCAGTCATGACAC GTCCAGGGAGTTGCGCAGGCTGCGC
FlagSigKF	AAGACAATTGAATGGACTACAA GGACGACGATGACAAGACCGGACCGCCACGGCTGAGC
SigKR	GGGAAGCTTACTCGAGCAGCTCAAAATCGG
RskAMtb_F	ATATATAAAGCTTATGACTGAACATACCGATTTTGAGC
RskAMtb_R	ATATGCGGCCGCTCACCCGAGCGGCAGCT
5′KOtriadYEF	GCA**GCGGCCG**CCGATTTTGCTGAAATTTG
P83YER	CATAATGTATTCCTGTAGGTTGAGGTG
P83LucF	CCTCAACCTACAGGAATACATTATGGAAGACGCCAA AAACATAAAG
LucXhoIR	GCA**CTCGAG**CCTACAATTTGGACTTTCC
SigKYeBspHIF	GGC**TCATGA**ATGAATGTTCTGTGGAAC
SigKYeXbaIR	GAG**TCTAGA**GTCATATTCGCATCTGTTTTTC
RskAYeXbaIR	ATG**TCTAGA**CTCCGGTGGAAAAAGTCTTAC
RskAL180*WAR	GAC**TCTAGA**CTTATTGTGTCGGCCCTTGAGTATTC
RskAW86*R	GGC**TCTAGA**TTAAGGGTTTCGCTTCA TGTGGCGGATATTGATCGGTGGTAACTGCAACTCCAG CCGTTTCCAAACGCGCTCAGGTGG
SigKC181SR	CATATTCGCATCTGTTTTTCATAAGCC TACAGAGTCCTTTAGGTGGTCC
SigKC181SF	GGACCACCTAAAGGACTCTGTAGGCTT ATGAAAAACAGATGCGAATATG
SigKYeNdeIF	GGCATATGGATGAATGTTCTGTGGAAC
SigKYeBamHIR	GGATCCTAGAGTCATATTCGCATCTGTTTTTC

(2) Plasmids coding for truncated versions of RskA: To construct all these plasmids a fragment of *rskA* from pMV::dyadbovis-Lx70 was removed after XhoI-HindIII digestion (Figure 1A), replaced by PCR products obtained using *Pfu* cloned polymerase (Stratagen Inc.) and digested with the same enzymes. These PCR products were obtained using Rv0444cOF and a specific reverse primer (Table 2). As an example, this was done using RskA1/121R which contained a HindIII site designed to introduce a stop codon in *rskA* after the 121 amino acids of RskA. In this case, the ligation of the PCR product obtained using *M. tuberculosis* gDNA template has generated pMV::dyad1/121Rv whereas the ligation of the PCR product obtained using the *M. bovis* gDNA template has generated pMV::dyad1/121bovis. Lastly, pMV::dyadΔrskA was obtained by self ligation of pMV::dyadbovis-Lx70 digested with XhoI-HindIII and treated with T4 polymerase.

(3) pOE-Lx70 versions of the plasmids: To increase our signal we generated new version of luciferase expressing plasmids where the *sigK-rskA* are under the control of *hsp60p*. We digested pMV::dyadbovis-Lx70 with PciI, treated with T4 DNA polymerase to generate blunt ends and further digested with HindIII. This product was ligated with *hsp60p* from pMV261 digested with DraI and HindIII to generate pOE-Lx70 where *hsp60p* has replace the dyad. In order to re-incorporate the dyads, amplified the *M. tuberculosis* version with SigK3F and Rv0444-HindIII primers (Table 2) and introduced it in pOE-Lx70 after a ligation of MunI-HindIII digestion products to obtain pOE-Lx70DyadRv. All the other versions of RskA were reintroduced in this backbone plasmid as described above using XhoI-HindIII.

(4) Plasmids expressing SigK with serines instead of cysteines: To generate these constructs, site-directed mutagenesis on cysteine amino acids residues position 133 and 183 on SigK was performed, using three fragments PCR assembly method and specific primers SigK133F, SigK133R, and SigK183R (Table 2). PCR product was digested by MunI-HindIII and cloned into pOE-Lx plasmid digested by the same enzymes to generate pOE-Lx SigKaloneSer. Then, *rskA* from *M. tuberculosis*, *M. bovis* and *M. orygis*, previously digested by XhoI-HindIII, were cloned into the generated pOE-Lx::DyadSerM.tb, pOE-Lx::DyadSerM.bo and pOE-Lx::DyadSerM.or.

(5) FLAG-tagged SigK: To generate the FLAG versions of SigK, we introduced a 1X FLAG-tag at the N-terminal part of *sigK* in both cysteine and serine version. Both versions of *sigK* were amplified from plasmids pOE-Lx::SigKalone and pOE-Lx::SigKaloneSer by PCR using *Phusion* HF DNA polymerase (New England, BioLabs) and primers FlagSigKF and SigKR (Table 2). PCR products and pOE-Lx plasmids were digested by MunI-HindIII and ligated together. Then, *rskA* versions from *M. tuberculosis*, *M. bovis* and *M. orygis* were amplified from previous pOE-Lx plasmids, digested by XhoI-HindIII and ligated to pOE-Lx::SigKalone and pOE-Lx::SigKaloneSer to generate pOE-Lx::SigKaloneFlag with Dyad*_*tuberculosis*_*, Dyad*_*bovis*_* and Dyad*_*orygis*_*. versus pOE-Lx::SigKaloneSerFlag with Dyad*_*tuberculosis*_*, Dyad*_*bovis*_* and Dyad*_*orygis*_*.

(6) *Nocardia* version of *sigK-rskA*: The locus from *Nocardia asteroides* 42007, *N. nova* 90960 or *N. abscessus* 91107 was amplified by the AccuPrime^TM^ Taq DNA polymerase High Fidelity (Invitrogen), using NfRv3416F and NfRv3417R primers. These PCR products were cloned into TOPO using TOPO^®^ TA Cloning^®^ Kit (Invitrogen). The fragments were sequenced at the McGill University and Genome Quebec Innovation Center, and the sequence of the *sigK* locus from *N. asteroides* was deposited in GenBank (accession numbers: FJ935781). The TOPO plasmid comprising the *N. asteroides* locus was digested using BsaBI, ligated with pMV::dyadRv-Lx70 digested with HindIII-ApaLI (which removed the dyad from *M. tuberculosis*) and treated with T4 polymerase to obtain pMV::dyadN42-Lx70. In a derivate plasmid called pMV::dyadN42stop-Lx70, a premature stop codon was inserted in *rskA*_*nocardia*_ by ligation of pre-annealed primers (PpuMIStopF and PpuMIStopR) with PpuMI digested pMV::dyadN42-Lx70.

All plasmids used in *Mycobacteria* (Table 1) were derived from pMV306 and were therefore integrated into the genomes of the specified mycobacteria. Plasmids were electroporated into *M. smegmatis*, *M. tuberculosis* H37Ra and H37Rv, and *M. marinum* as previously described (Belley et al., 2004). Hygromycin resistant clones were verified by PCR and selected for further experiments.

### Luciferase Assays for Mycobacteria (LuxAB)

As described before (Veyrier et al., 2008), bacteria were grown in 7H9 with hygromycin until the cultures reached an OD_600_ of 0.5. After centrifugation, cultures were resuspended in Phosphate-Buffered Saline (PBS) and 10 μl of 1% n-decyl aldehyde (Sigma) in ethanol was added to 90 μl (9 × 10^6^ bacteria) in 96 well plates (Becton Dickinson labware). Light was immediately measured in the Victor 3^TM^ Wallac 1420 Multilabel Counter (PerkinElmer) over a 10 s period. For each experiment, 2 transformants have been measured two times and luminescence output was expressed in RLU (Relative Light Unit).

To test expression of luciferase in bacteria harboring the transposon, bacteria were grown overnight in 1 ml of 7H9 with hygromycin and kanamycin. After centrifugation, cultures were resuspended in Phosphate-Buffered Saline (PBS) and tested as described above. In this case, we normalized the amount of light with the OD_600_ measured in the 96 well plates with the Victor 3^TM^ Wallac 1420 Multilabel Counter (PerkinElmer).

### RNA Extraction and Quantitative RT-qPCR

The aim was to determine the expression of *sigK*_*tuberculosis*_ in *M. smegmatis* harboring the different plasmids. To do that we performed RT-qPCR and normalized against the RNA amount of *sigA*_*smegmatis*_ (Manganelli et al., 1999). RNA was extracted from cultures with an OD_600_ of 0.4 to 0.6 by a modified phenol-chloroform extraction method (Charlet et al., 2005). The RT-qPCR methods as well as the primers used for *sigK* have already been described (Charlet et al., 2005) except for the primers (SigAMsF and SigAMsR) used for *sigA*_*smegmatis*_ which are listed in Table 2.

### Protein Preparation and Immunoblot

For secreted protein (MPT70), 10 ml of culture supernatants of indicated *M. tuberculosis* strains were concentrated using a Millipore Ultra-10 Centrifugal Filter Unit (10 000 MW cut-off) and filtered through a 0.22 μm membrane. For membrane associated proteins (MPT70 and MPT83), culture pellet of a 10 ml culture was resuspended in 1 ml of water and samples were subjected to FastPrep (BIO 101 Savant) for 15 s at 6.0 rpm 3 times. To verify the expression of SigK, single colonies from *M. smegmatis*::*sigK, M. smegmatis*::*dyadtub, M. smegmatis*::*dyadbov*, and *M. smegmatis*::*dyadoryx*, as well as its mutated versions (C133S and C183S) were picked, inoculated into 2 mL of 7H9 medium supplemented with 50 μg mL^–1^ hygromycin and incubated 48 h at 37°C, 250 rpm. Bacteria harvesting was done by centrifugation of 1 mL of culture at 8,000 × *g*, RT, 10 min. Cell pellets were resuspended in 200 μL 1X PBS. Lysis was performed by addition of 65 μL of 4X Laemmli sample buffer, followed by boiling at 100°C for 10 min. Samples were run on a 12% SDS−PAGE gel for 1 h at 200V.

For blotting assay, protein transfer was performed on PVDF membranes (BioRad) at 80 V for 2 h. Membrane blocking was carried out for 1 h at room temperature, using TBS-Tween 0.1% containing 5% BSA (TBS-TB). Incubation with rabbit polyclonal antibodies against MPT70 and MPT83 (gifts of Harald Wiker) (1:500 dilution), mouse monoclonal antibodies against HSP65 (Abcam), Mouse monoclonal DYKDDDDK Tag Antibody (FG4R) and Mouse RpoB Monoclonal Antibody (8RB13) were blotted onto the PVDF membrane overnight at 4°C followed by 1h incubation with anti-rabbit IgG − horseradish peroxidase conjugate (1:15 000 dilution) or anti-mouse IgG-horseradish peroxidase conjugate (1:15 000 dilution). Three washing steps with TBS-Tween 0.1% of 10 min each were added after incubation with both antibodies. Proteins were visualized by ECL, using the substrate SuperSignal West Pico (Thermo Fisher Scientific).

### *Enterobacterales* Plasmids Construction

(1) pKO5′Luc: This plasmid, with a pHS1 (a temperature-sensitive derivative of pSC101) origin of replication, was used to express the firefly luciferase under the control of *mpt83*p from *Y. enterocolitica*. To construct this plasmid the luciferase was fused to *mpt83*p by a 3 step PCR using the primers P83LucF/LucXhoIR to amplify the luciferase and 5′KOtriadYEF/P83YER (Table 2) to amplify the promoter. The fused PCR product were digested with NotI-XhoI and cloned in pKO3 (Link et al., 1997) digested with NotI-SalI to give pKO5Luc.

(2) pBAD::Km with *sigK* and *rskA* from *Y. enterocolitica*: Coding sequences for SigK, SigK-RskA, SigK-RskA179 and SigK-RskA85 were amplified using specific primers. In all cases, the forward primer was SigKYeBspHIF, and the reverse primers were SigKYeXbaIR, RskAYeXbaIR, RskAWA179^∗^R, and RskAW85^∗^R, respectively. Restriction sites for BspHI (forward primer) and XbaI (reverse primer) were used to clone into pBAD digested with NcoI and XbaI.

(3) pBADSigKmut: For SigKC181S mutant, *sigK* was amplified using the forward primer mentioned above, and SigKC181SR. Different variants of *rskA* were amplified using the reverse primers already described, and the forward primer SigKC181SF, which overlaps with SigKC181SR, allowing the fusion with *sigK*C181S and generating the variants *sigK*C181S*rskA*, *sigKC*181S-*rskA*179 and *sigK*C181S-*rskA*85. The PCR products were digested with BspHI and XbaI and cloned into pBAD-Km vector digested with NcoI and XbaI.

(4) pET28a::SigK: SigK was cloned into pET28a(+) vector using primers SigKYeNdeIF and SigKYeBamHIR. Both PCR product and vector were digested by NdeI and BamHI for introduction of a N-terminal 6xHis. After ligation, constructs were cloned in *E. coli* DH5α and then transformed in *E. coli* BL21(DE3) for protein expression.

### Luciferase Assays for *Enterobacterales* (Firefly Luciferase)

For the in vitro luciferase assay, bacteria co-transformed with pBAD-Km vector containing the different SigK-RskA constructions and pKO5′luc, were grown overnight at 30°C, 200 rpm, in LB medium containing 50 μg mL^–1^ kanamycin and 25 μg mL^–1^ chloramphenicol. From the overnight cultures, 100 μL were inoculated into 3 mL LB with the antibiotics already mentioned. In a 96 well microplate, 67.5 μL of each fresh culture were dispensed in triplicate considering two conditions: induction and non-induction. For induction condition, 0.06% L-arabinose was added. Therefore, the plate included bacteria expressing the different versions of SigK-RskA (wild-type and mutant), as well as bacteria expressing the empty vector (pBAD-Km, as a negative control), both in absence and presence of L-arabinose. The induction was carried out for the indicated time at 30°C. Then, 75 μL of the Luciferase Assay Reagent (Promega) were added in each well, and the luminescence was measured using a Wallac Victor 3 (Perkin Elmer) luminometer. Luminescence was normalized by the bacterial OD determined by absorbance at 600 nm.

### SigK Overexpression and Purification From *E. coli*

From an overnight culture, 10 mL were inoculated into 1 L of LB medium supplemented with 50 μg mL^–1^ kanamycin and grown at 37°C, 250 rpm until reaching an OD_600_ of 0.6. Induction was performed with 1 mM isopropyl β-D-1-thiogalactopyranoside (IPTG) at 37°C for 4 h. Bacteria harvesting was carried out by centrifugation at 10,000 rpm, 4°C, 15 min. Cell pellets were resuspended in 20 mL native binding buffer (50 mM NaH_2_PO_4_, 300 mM NaCl, and 10 mM imidazole, pH 8). Pellets were kept on ice, treated with 1 mg mL^–1^ lysozyme and 1 mM PMSF for 30 min, and lysed by sonication. Lysates were clarified by centrifugation at 15,000 rpm, 4°C, 30 min, and purified using HisPur^TM^ Ni-NTA Resin (Thermo Fisher Scientific). Lysates were loaded onto the purification column (previously equilibrated with native binding buffer), unspecific binding was removed by washing (50 mM NaH2PO4, 300 mM NaCl, and 20 mM imidazole), and proteins were eluted (50 mM NaH2PO4, 300 mM NaCl, and 150 mM imidazole). Protein identity was confirmed by western blotting using a monoclonal Anti-His IgG1 − from mouse (Sigma).

### SigK Western Blotting

To verify the expression of SigK, single colonies from *E. coli* BL21(DE3)::*sigK, E. coli* BL21(DE3)::*sigK-rskA, E. coli* BL21(DE3)::*sigK-rskA182* and *E. coli* BL21(DE3)::*sigK-rskA81* were picked, inoculated into 5 mL of LB supplemented with 50 μg mL^–1^ kanamycin and incubated overnight at 37°C, 250 rpm. The overnight cultures were diluted into 500 mL LB supplemented with 50 μg mL^–1^ kanamycin, and incubated at 37°C, 250 rpm, until OD_600_ reached 0.6. Induction was performed as described before. Bacteria harvesting was done by centrifugation at 10,000 rpm, 4°C, 15 min. Cell pellets were resuspended in 10 mL 1X PBS containing 0.5% SDS, kept on ice and lysed by sonication. Lysates were clarified as previously indicated. Then, 5 μL of each sample were diluted into 7 μL H_2_O and 4 μL of 4X Laemmli sample buffer (0.2 M Tris pH 6.8, 0.08% w/v SDS, 0.2% v/v β-mercaptoethanol, 0.4% v/v Glycerol, 0.002% w/v Bromophenol blue), and boiled at 100°C for 10 min. Samples were run on a 12% SDS−PAGE gel for 1 h at 200 V.

For blotting assay, protein transfer was performed on PVDF membranes (BioRad) at 90 V for 1 h 30 min. Membrane blocking was carried out for 1 h at room temperature, using TBS-Tween 0.1% containing 5% BSA (TBS-TB). Incubation with 1:500 in TBS-TB of primary antibody (monoclonal Anti-His IgG1 − from mouse) was performed overnight at 4°C. An anti-mouse IgG − HRP conjugate − from rabbit was used as a secondary antibody (1:10000 in TBS-TB), incubating for 1 h at RT. Three washing steps with TBS-Tween 0.1% of 10 min each were added after incubation with both antibodies. Proteins were visualized by ECL, using the substrate SuperSignal West Pico (Thermo Fisher Scientific).

RpoB detection [by incubation with an RNA polymerase beta Monoclonal Antibody (8RB13) – from mouse], was used as loading control.

### SigK and RskA *in silico* Study

The sequences of SigK from *M. tuberculosis* and *Yersinia enterocolitica* were added to a database constituted by another study and containing 4352 sequences of ECF Sigma factors (Pinto and da Fonseca, 2020). CD-HIT version 4.8.1 (Fu et al., 2012) was used to keep only one representative sequence for those sharing 95 % or more of identity. The resulting 3717 sequences were aligned using MAFFT version 7.453 using 25 refinement iterations (Katoh and Standley, 2013). A phylogenetic reconstruction was then performed using FastTree version 2.1.11 (Price et al., 2010) with the LG+CAT model. Finally, the tree was visualized using FigTree version 1.4.3^2^.

The sequences of representative genomes of the RefSeq database have been downloaded (Supplementary Table S1). Subsequently, sequences homologous to SigK were searched in the set of coding sequences using BLASTP version 2.10.0. The sequences having an e-value and a percentage of similarity greater than or equal to 1e-10 and 50%, respectively, were retained. The RskA homologous sequences were also searched by BLASTP, with the default parameters, but with the obligation that the RskA be adjacent to the identified SigK, considering the polarity of the genes. In the event that no RskA was found, the adjacent gene was still extracted and investigated manually by annotation and COG categorization using EggNOG mapper (Huerta-Cepas et al., 2017).

The 4157 SigK sequences were then aligned by MAFFT with 25 iterations. The amino acids at positions 133 and 183 according to the sequence of *M. tuberculosis* were extracted in order to verify the conservation of the cysteines. Different sequence characteristics, such as the number of total cysteines, CXXC (zinc finger) and length were determined using a custom python script. Finally, the nucleotide sequences of the RskAs encoded as pseudogenes in RefSeq for the genus *Nocardia* have been downloaded, translated into amino acids and their length calculated with an in-house script.

## Data Availability Statement

The datasets presented in this study can be found in online repositories. The names of the repository/repositories and accession number(s) can be found in the article/Supplementary Material.

## Author Contributions

FV and MB designed the study. FV, CN, LL, HT, and AV performed and analyzed the experiments. FV, CN, AV, and MB wrote and edited the manuscript. All authors have read and approved the manuscript.

## Conflict of Interest

The authors declare that the research was conducted in the absence of any commercial or financial relationships that could be construed as a potential conflict of interest.

## Footnotes

2 http://tree.bio.ed.ac.uk/software/figtree/

## Supplementary Material

FIGURE S1 Alignment **(A)** and schematic representations **(B)** of RskA versions used in this study (natural or engineered variants).

FIGURE S2 **(A)** Effect of FLAG-tag linked to the N-terminus part of SigK was measured by luciferase under the control of *mpt70p* in *M. smegmatis* **(B)** Effect of His-tag linked to the N-terminus part of SigK was measured by luciferase under the control of *mpt83p* in *E. coli*. Error bars represent standard deviations of 3 independent experiments with 2 different clones each time.

FIGURE S3 Expression of SigK regulon through MPT70 antigen production was detected by immunoblot of *M. tuberculosis* ΔsigK cell extract. Dyad M.tb cysteines vs. serines version; Dyad M.bo cysteines vs. serines version; Dyad M.or cysteines vs. serines version.

TABLE S1 Table presenting the *in-silico* analyses of SigK homologues using RefSeq database.
