# Supplementary material for: RskA Is a Dual Function Activator-Inhibitor That Controls SigK Activity Across Distinct Bacterial Genera
Source: Front Microbiol. 2020 Sep 9;11:558166. doi: 10.3389/fmicb.2020.558166 (PMC7509140; doi:10.3389/fmicb.2020.558166)
Supplement: FIGURE S1 — Alignment (A) and schematic representations (B) of RskA versions used in this study (natural or engineered variants). [file Data_Sheet_1.DOCX]

***Supplementary material***

**Supplementary Figures**

**
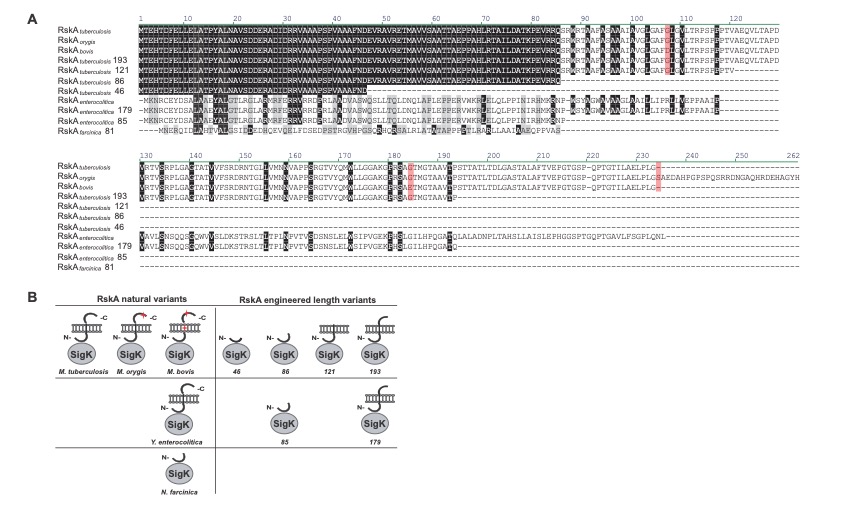
**

**Figure S1.** Alignment A) and schematic representations B) of RskA versions used in this study (natural or engineered variants).


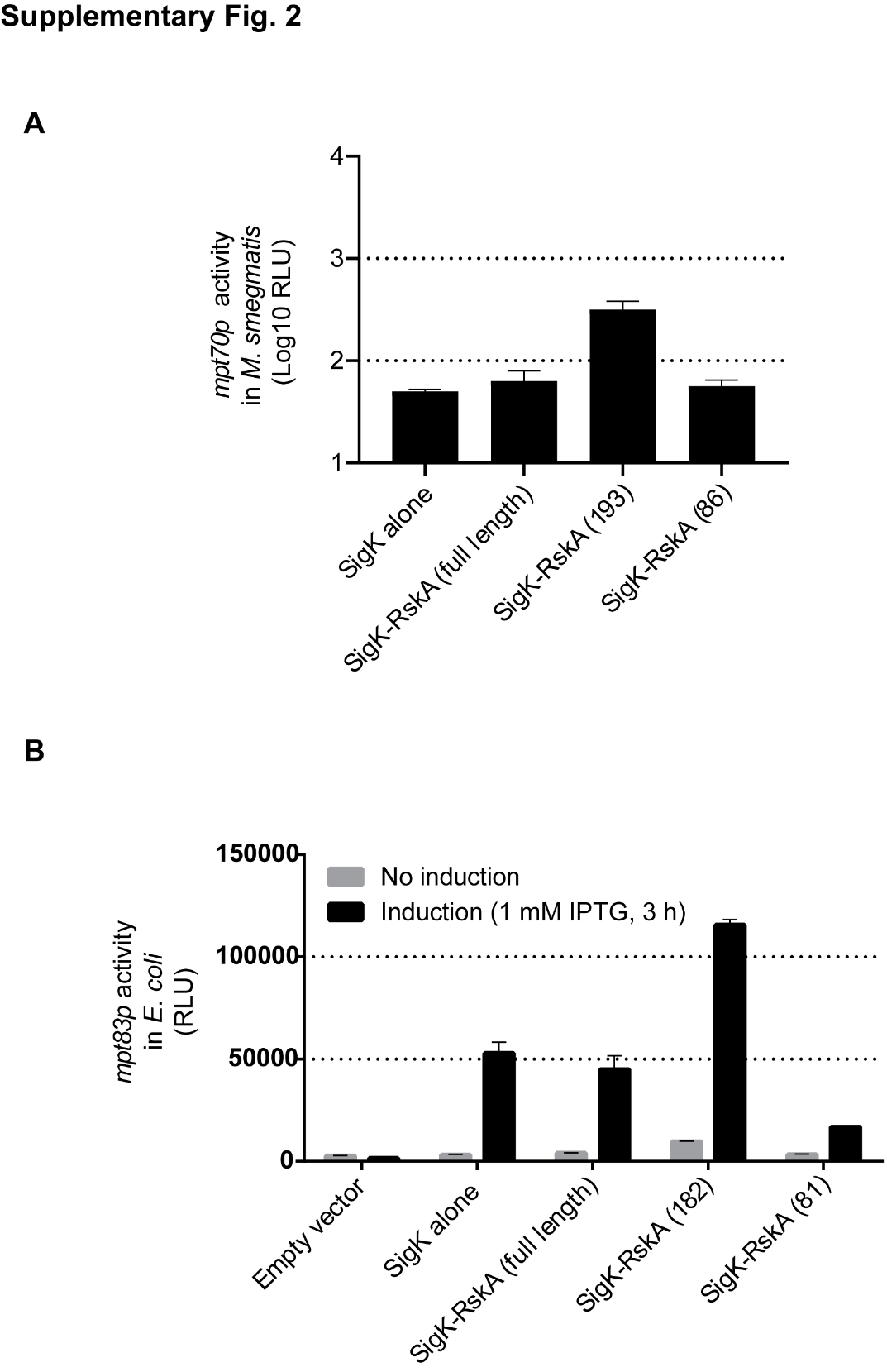


**Figure S2. A.** Effect of FLAG-tag linked to the N-terminus part of SigK was measured by luciferase under the control of *mpt70p* in *M. smegmatis* **(B)** Effect of His-tag linked to the N-terminus part of SigK was measured by luciferase under the control of *mpt83p* in *E. coli*. Error bars represent standard deviations of 3 independent experiments with 2 different clones each time.

**Figure S3:** Expression of SigK regulon through MPT70 antigen production was detected by immunoblot of *M. tuberculosis* ΔsigK cell extract. Dyad M.tb cysteines vs. serines version; Dyad M.bo cysteines vs. serines version; Dyad M.or cysteines vs. serines version.
